# Rationale and design of a multicenter randomized clinical trial of vestibulodynia: understanding pathophysiology and determining appropriate treatments (vestibulodynia: UPDATe)

**DOI:** 10.1080/07853890.2022.2132531

**Published:** 2022-10-21

**Authors:** Erin T. Carey, Elizabeth J. Geller, Andrea Rapkin, Debbie Farb, Haley Cutting, Jasmyn Akaninwor, Christopher Stirling, Andrey Bortsov, Steven McNulty, Peter Merrill, Pearl Zakroysky, Jesse DeLaRosa, Sheng Luo, Andrea G. Nackley

**Affiliations:** a Department of Obstetrics and Gynecology, University of North Carolina at Chapel Hill, Chapel Hill, NC, USA; b Department of Obstetrics and Gynecology, David Geffen School of Medicine, University of California at Los Angeles, Los Angeles, CA, USA; c Department of Anesthesiology, Center for Translational Pain Medicine, Duke University School of Medicine, Durham, NC, USA; d Duke Clinical Research Institute, Duke University, Durham, NC, USA; e Department of Biostatistics and Informatics, Duke University, Durham, NC, USA; f Department of Pharmacology and Cancer Biology, Duke University School of Medicine, Durham, NC, USA

**Keywords:** Vulvodynia, provoked vestibulodynia, pelvic pain, chronic overlapping pain conditions, microRNA, cytokine, biomarker, tricyclic antidepressants, nortriptyline, topical lidocaine, clinical trial

## Abstract

**Background:**

Limited data are available to establish evidence-based management protocols for vestibulodynia (VBD), a chronic vulvar pain condition that affects approximately 14 million women in the U.S. For the purposes of the study, our group subdivided VBD subtypes that may benefit from different types of treatment: 1) VBD peripheral (VBD-p), characterized by pain localized to the vulvar vestibule and 2) VBD central (VBD-c), characterized by VBD alongside one or more other chronic overlapping pain conditions (e.g. irritable bowel syndrome, temporomandibular disorder, and fibromyalgia syndrome) that affect remote body regions. Here, we describe the rationale and design of an NIH-funded multicenter clinical trial comparing the effectiveness of topical and/or systemic medication for alleviating pain and normalizing pain- relevant biomarkers among women with VBD-p and VBD-c.

**Methods:**

Participants will be randomly assigned to one of four parallel arms: peripheral treatment with 5% lidocaine + 0.5 mg/ml 0.02% oestradiol compound cream + oral placebo pill, 2) central treatment with the tricyclic antidepressant nortriptyline + placebo cream, 3) combined peripheral cream and central pill treatments, or 4) placebo cream and placebo pill. The treatment phase will last 16 weeks, with outcome measures and biomarkers assessed at 4 time points (0, 8, 16, and 24 weeks). First, we will compare the efficacy of treatments in alleviating pain using standardized tampon insertion with a numeric rating scale and self-reported pain on the short form McGill Pain Questionnaire. Next, we will compare the efficacy of treatments in improving perceived physical, mental, and sexual health using standardized questionnaires. Finally, we will measure cytokines and microRNAs in local vaginal and circulating blood samples using multiplex assays and RNA sequencing, and determine the ability of these biomarkers to predict treatment response.

**Conclusion:**

This is the first multicenter randomized controlled trial to evaluate the efficacy of peripherally and centrally acting medications currently used in clinical practice for treating unique VBD subtypes based on distinct clinical and biological signatures.

**Administrative information:**

Vestibulodynia UPDATe is a multi-centre, two-by-two factorial designed randomized, double-blind, placebo-controlled trial registered at clinical trials.gov (NCT03844412). This work is supported by the R01 HD096331 awarded to Drs. Nackley, Rapkin, Geller and Carey by the Eunice Kennedy Shriver National Institute of Child Health and Human Development (NICHD).

**Key messages**
- Peripheral lidocaine and oestradiol and centrally-targeted nortriptyline medications are used for the treatment of pain in women with VBD, but there is a lack of data from well-powered RCTs.
- This two-by-two factorial RCT will test the efficacy of these medications in VBD subtypes characterized by distinct clinical characteristics and biomarker profiles.
- We hope that results will provide clinicians with scientific evidence of therapeutic efficacy in distinct VBD subtypes in an effort to direct and optimize treatment approaches.

## Introduction

Vestibulodynia (VBD) is the most common cause of sexual pain, affecting 16% of reproductive aged women in the United States [1]. The pain is chronic, lasting over three months, and compromises psychological functioning, interpersonal relations, and daily activity [2]. As further evidence of its public health significance, VBD and related vulvar pain conditions cost the US economy over $70 billion annually [3]. Despite its high prevalence and significant economic burden, VBD remains ineffectively treated due to its unclear aetiology and heterogeneous clinical presentation. Some women present with localized provoked pain restricted to the vaginal vestibule and others present with VBD and chronic overlapping pain conditions (COPCs) such as irritable bowel syndrome (IBS; 35%) [4] temporomandibular disorder (TMD; 78%) [5] and fibromyalgia syndrome (FMS; 17%) [4]. In the absence of data to guide treatment approaches for VBD patients with diverse symptomology, many different therapies are used on a trial-and-error basis which can delay effective care [6].

In 2015 International Society for the Study of Vulvovaginal Disease, the International Society for the Study of Women’s Sexual Health, and the International Pelvic Pain Society developed a consensus statement for descriptors of women with vulvodynia to classify the distribution, onset and timing of pain. As the neuropathophysiology of vulvodynia is complex and characterized by both peripheral and central sensory abnormalities, we opted to divide these women into two groups based on presence of COPCs for study purposes [7]. The VBD peripheral (VBD-p) subtype is characterized by localized pain specific to the vaginal vestibule, and the VBD central (VBD-c) subtype is characterized by pain at vestibular and remote body regions [8]. Women with VBD-p frequently exhibit normal psychological profiles; balance in circulating pro- and anti-inflammatory cytokines; and dysregulation in microRNAs that regulate the expression of genes in oestrogen pathways [8]. In contrast, women with VBD-c report decreased functional status and increased somatic awareness; lack compensatory increases in anti-inflammatory cytokines; and have dysregulation in microRNAs that regulate the expression of genes relevant to muscle, nerve, and immune cell function [8]. These data suggest that VBD-p and VBD-c have unique aetiologies (localized pain with peripheral neurosensory disruption versus widespread pain with a central sensory contribution) and may, therefore, respond differently to peripheral and centrally-targeted treatments.

Local anaesthetics and oestrogen are among the most commonly prescribed peripheral treatments [6,9,10]. Topical 5% lidocaine for 7 weeks has been shown to decrease dyspareunia in women with VBD [11], likely due to its ability to block the activity of sodium channels on peripheral nociceptors and prevent the transmission of pain to the CNS [12,13]. Topical oestrogen for 3 months has been shown to reduce peripheral pain and inflammation [14] as well as improve sexual function for premenopausal women with vulvar pain [15]. Oestrogen plays a critical role in trophic support and neuroplasticity of vaginal tissues, with deficient oestrogen levels resulting in atrophy and increased innervation of pain-sensing nerve fibres [16,17]. Further evidence that women with VBD may be deficient in oestrogen come from the Wesselmann study demonstrating that tampon-induced vaginal pain is highest in the premenstrual phase, corresponding with systemic low oestrogen levels [18], and our own work, where we demonstrated that women with VBD-p have increased expression of microRNAs that negatively regulate transcripts vital for oestrogen signalling [8]. Lidocaine and oestrogen are often compounded into a combined cream that can produce a synergistic analgesic effect [9]. Yet, data from well-powered RCTs in support of these treatments are lacking.

Tricyclic antidepressants (TCAs) are the most commonly prescribed centrally-targeted treatment for the management of vulvar pain [10] and have been shown to improve sexual function [19]. An open trial of nortriptyline for 2 months found that 6/7 women had a complete or partial reduction in chronic pelvic pain [20]. In a case study, nortriptyline completely alleviated vaginal pain in a woman for whom topical lidocaine, oestrogen, and other peripheral treatments failed [21]. While the TCA Amitriptyline has been prescribed for vulvar pain, clinical studies demonstrating its efficacy are limited and upwards of 30% of women may discontinue its use due to sedation and other side effects [22]. The anti-epileptic Gabapentin has also been commonly prescribed for vaginal pain, however recent results from a randomized controlled trial found that gabapentin was ineffective in reducing pain among women with VBD [23]. Nortriptyline has been well studied for the treatment of other neuropathic pain disorders and evidence-based guidelines support its use as a first-line medication [24]. Nortriptyline produces analgesia via multiple molecular mechanisms in the CNS, including (1) inhibiting reuptake of norepinephrine to promote descending inhibition [25], (2) blocking sodium channels to inhibit the activity of nociceptive neurons [26], and (3) inhibiting the release of pro-inflammatory cytokines from glia so as to reduce neuroinflammation [27].

However, as concluded in recent evidence-based literature reviews, interpretation of treatment success remains difficult due to the limitations of published studies, including poor patient selection, lack of adequate controls, and limited follow-up data [19,28]. Thus, there is a critical need for randomized controlled trials (RCTs) to evaluate the efficacy of these peripheral and centrally-targeted treatments, especially among women with different VBD subtypes.

Thus, we are conducting new multidisciplinary studies that plan to identify effective strategies for the management of VBD. We will also continue to characterize VBD subtypes with data obtained from this proposed multi-site RCT. We will compare the efficacy of peripheral, central, and combined treatments in alleviating objectively-measured pain and improving patient reported outcomes for women with VBD. In addition, we will evaluate cytokine and microRNA biomarkers that help determine underlying pathophysiology and predict treatment response. To evaluate these measures, we will apply a randomized, double-blinded, placebo-controlled factorial study design to evaluate the analgesic efficacy of: (1) peripheral treatment with lidocaine/oestradiol compound cream, (2) centrally-targeted treatment with the oral tricyclic nortriptyline, or (3) combined peripheral and central treatment in women with newly diagnosed VBD. We hypothesize that women with VBD-p will exhibit increased levels of inflammatory mediators in the vaginal vestibule and preferentially respond to peripheral lidocaine/oestradiol treatment, while women with VBD-c will exhibit increased levels of inflammatory mediators in blood and preferentially respond to centrally-targeted or combined treatment. With this study we hope to advance our knowledge of the pathophysiologic mechanisms underlying VBD-p and VBD-c, determine the efficacy of peripheral, central, and combined therapies in alleviating pain in women with VBD-p and VBD-c, and identify biomarkers that predict treatment response.

## Patients/materials and methods

### General study design

Vestibulodynia UPDATe is a multi-centre, two-by-two factorial designed randomized, double-blind, placebo-controlled trial registered at clinical trials.gov (NCT03844412) designed to determine the efficacy of two treatments, alone or in combination, compared to matching placebo. Women will be recruited from two study sites and complete a 16-week treatment phase, with outcome measures and biomarkers assessed at four time points (0, 8, 16, and 24 weeks). The primary endpoints include change in pain, self-reported health, and cytokine/microRNA measures at 16 weeks, with secondary endpoints of change in these measures at 8 weeks and 24 weeks. Participants will be randomly assigned to one of four treatment groups: 1) peripheral treatment in the form of lidocaine/oestradiol compound cream + oral placebo pill, 2) centrally-targeted treatment in the form of placebo cream + oral nortriptyline pill, 3) combined treatment in the form of lidocaine/oestradiol compound cream + oral nortriptyline pill; or 4) placebo cream and placebo pill. A total of 400 women (200 per site) with VBD diagnosis confirmed by one of the study clinicians will be recruited from 4 November 2019 to 1 December 2023 from the University of North Carolina (UNC) and the University of California Los Angeles (UCLA) under reliance of the single institutional review board (IRB) protocol at Duke University. Eligible women will participant in the study for 24 weeks with a total of four study visits.

### Study population

The study population is comprised of premenopausal and perimenopausal women with VBD of any ethnicity or race. Based on historical patterns of recruitment, we expect to enroll 10% Hispanic/Latino and 90% non-Hispanic/Latino women. Of the non-Hispanic/Latino women, we expect the racial distribution will be 70% White, 10% Black, 5% Asian and 5% other groups. This distribution is similar to that among people reporting VBD in other U.S. demographic populations in other clinical trials [29].

#### Inclusion criteria

Women 18-50 years old with greater than three months of pain who report pain level of 3 or greater on the 11-point 0-10 numeric rating scale with insertional sexual activity and/or with touch to the vaginal opening, and/or who report pain level of 3 or greater with tampon insertion/removal at the first study visit.

#### Exclusion criteria

Women with active vulvar conditions which result in pain of the vulva including active skin infections, dermatoses, fissures and vaginal infections through patient report and physical inspection are excluded. We are also excluding women with obvious signs of vaginitis or vaginosis such as yellow green or white thick clumpy discharge. These women will be rescheduled if wet mount is unavailable on the day of their study visit. Other exclusions include untreated atrophic vaginal tissue, prior vestibulectomy, pregnancy or at risk for pregnancy, postpartum or breastfeeding, any uncontrolled medical condition (e.g. renal impairment, hematological disease, cardiovascular disease, hepatic insufficiency, neurological disorder, autoimmune disease, or respiratory illness) or uncontrolled psychiatric disorder, history of intolerance to nortriptyline, topical lidocaine or topical oestradiol. Also excluded are postmenopausal women, those with systemic inflammation (e.g. morbid obesity, systemic lupus erythematosus), recent cancer diagnosis or treatment, current or recent use (within the past 1–3 months, drug dependent) of monoamine oxidase inhibitors (MAOIs), and some selective serotonin reuptake inhibitors (SSRIs), serotonin-norepinephrine reuptake inhibitors (SNRIs), and norepinephrine-dopamine reuptake inhibitors (NDRIs). Women with generalized vulvodynia alone in the absence of VBD are excluded. Women actively enrolled in pelvic floor physical therapy or those who received botulinum toxinA injections to the pelvic floor muscles in the last 12 months, or pelvic nerve blocks in the last 3 months are excluded. Detailed inclusion and exclusion criteria are listed in Table 1.

**Table 1.** Study inclusion and exclusion criteria.

Inclusion criteria
Female age 18–50 years
English-literate BMI under 40
Willingness to provide informed consent
>3 continuous months of insertional dyspareunia, pain to touch or tampon insertion
Average pain >3 on a 0–10 numeric rating scale with the tampon test at first study visit, reports pain with sexual contact in the past 3 months involving penetration into the vagina, or pain with touch to the vaginal opening
Exclusion criteria
Presence of active dermatologic vulvar disease or vaginal infection
Untreated atrophic vaginitis (participants may undergo treatment with topical hormone therapy for a minimum of six weeks to be considered for re-evaluation for enrolment)
Previous vestibulectomy – full or partial
Pregnancy or at risk for pregnancy during the study period
Within the first six months postpartum
Currently breastfeeding/lactating, or within 3 months of discontinuing breastfeeding/lactation
Active incarceration
Cancer diagnosis within the past year
Chemotherapy and/or radiation treatment within the past year
Noncontrolled medical condition: significant renal impairment, hematological disease, hepatic insufficiency, cardiovascular disease (cardiac conduction disturbance, congestive heart failure, hypertension), neurological disorder, autoimmune disease, or respiratory illness
Clear inflammatory states (e.g. Rheumatoid Arthritis, Lupus, Ulcerative Colitis, Crohn’s disease, Ehlers-Danlos Syndrome)
Use of immunosuppressant medications
History of intolerance to nortriptyline, topical lidocaine, or topical oestradiol
Contraindications to use of nortriptyline: current use, or use within the past 1-3 months, of MAOIs, some NDRIs, Ethylphenidate (DPH), MDPV (Methylenedioxypyrovalerone), Pipradrol (Meratran), Prolintane (Catovit/ Promotil), myocardial infarction in past year, active psychotic or suicidal thoughts, narrow angle closure glaucoma
Discontinuation of medications for 30-90 days, depending on the medication type, dosage, frequency, duration, and half-life. All concomitant medications will need to be documented at the time of screening so the Principal Investigator and medical doctors at each participating site can determine a conservative window. Medications include, but are not limited to: topical lidocaine, oestradiol, or lidocaine/oestradiol to the vulvar vestibule, nortriptyline or other TCAs, pregabalin, and gabapentin.
Contraindications to the use of lidocaine or local anaesthetics
Contraindications to the use of topical oestrogen therapy
Post-menopausal status, defined as no menses for 12 consecutive months or surgical removal of both ovaries. (Hysterectomy is not an exclusion)
Botulinum toxin A injections of the pelvic floor muscles in the last 12 months, or pelvic nerve blocks in the last three months
Ongoing pelvic floor physical therapy
Not currently enrolled or planning to enroll in another clinical trial during the course of the trial

### Recruitment, screening, and enrollment

Primary recruitment will occur in the clinic setting in both subspecialty pelvic pain clinics and other specialty gynaecologic clinics. Additional recruitment strategies will include flyer distribution, social media posts, and mass email blasts to UNC and UCLA students and employees every three months. Study information will be placed online on a study-specific website (sites.duke.edu/updatestudy), social media sites (e.g. Instagram, Facebook, Twitter), relevant University (e.g. Duke List) and Association (e.g. National Vulvodynia Association) sites, and clinicaltrials.gov. In addition, we will use study sites’ data warehouses to identify potential participants with VBD-associated ICD-9 or ICD-10 codes.

Screening will be conducted by telephone call, by email, or in-person at a clinic visit (please see the Appendix for the phone/email screener). The purpose of the study, study interventions and evaluations, and the potential risks and benefits of participation, will be explained to eligible participants. If a potential participant meets all inclusion/exclusion criteria during the in-person screening verification at the beginning of Visit 1, she will be provided the site-specific Institutional Review Board (IRB)-approved informed consent form to review (please see the Appendix for UNC and UCLA consent forms). If she then agrees to participate, she will sign the consent form and complete a pregnancy test and tampon test. The urine pregnancy test will be completed according to manufacturer’s instructions. The tampon test and Q-tip test will be completed as described in the Outcome Measures section below. In addition, she will be asked to rate her pain with sexual contact involving penetration into the vagina and pain with touch to the vaginal opening on a 0-10 numeric rating scale.

Participants may be enrolled if they (1) sign the informed consent, AND ‘(2) meet all inclusion/exclusion criteria, AND (3) have a negative pregnancy test result, AND rate pain as ≥3/10 with the tampon test, with sexual contact involving penetration into the vagina, and/or with touch to the vaginal opening.

### Study medications

Participants will be randomly assigned a combination of either (1) peripheral treatment in the form of 5% lidocaine/0.5 mg/ml 0.02% oestradiol compound cream + placebo pill, (2) centrally-targeted treatment in the form of placebo cream + nortriptyline oral pill (up to 50 mg or highest tolerated dose), (3) combined treatment in the form of 5% lidocaine/0.5 mg/ml 0.02% oestradiol compound cream + nortriptyline; or (4) placebo cream + placebo pill. Participants will be prompted weekly by email to enter their use of medications in a REDCap calendar.

Lidocaine/oestradiol cream will be used to target peripheral nerves and tissues affected in VBD; and the comparison treatment will be an identical-appearing placebo Moisturel™ cream. The cream has a dose-controlled pump action that delivers a predetermined dose of medication. Participants will be provided with a diagram and written instructions detailing how to apply the active or placebo cream to the vaginal vestibule daily for weeks 1–16. Treatment with lidocaine/oestradiol or placebo cream will be terminated at 16 weeks.

Nortriptyline is a centrally-acting tricyclic antidepressant that is FDA-approved for treatment of neuropathic pain. Dosing will begin with one 10 mg pill nightly for week 1, then two 10 mg pills nightly for week 2, three 10 mg pills nightly for week 3, four 10 mg pills nightly for week 4, and five for weeks 5–16. A slow titration period is provided to reduce side effects. In the event of side effects, participants will be advised to decrease dosage by one pill weekly until a tolerable dose is achieved. Treatment with oral nortriptyline or placebo pill will be tapered off over weeks 16–18, decreasing the dose by 10 mg every four days. Participants will be provided with a list of drugs to avoid that have the potential to interact with nortriptyline.

### Randomisation and blinding

Drug assignments will be determined by the Duke Clinical Research Institute (DCRI) using randomized blocks, and then stratified by enrollment site. The DCRI will create a SAS program to generate the random sequence that will be loaded in Duke’s Research Electronic Data Capture (REDCap).

Participants and research personnel will be blinded to medication assignment. Blinding of the study, with respect to treatment groups will be preserved by the use of placebo cream and capsules that are identical in appearance to the active study drugs. Investigators may be asked at the end of the trial if they had obtained any information that may have led to the potential unblinding of treatment.

### Assessment of outcome measures

**Figure 1.**
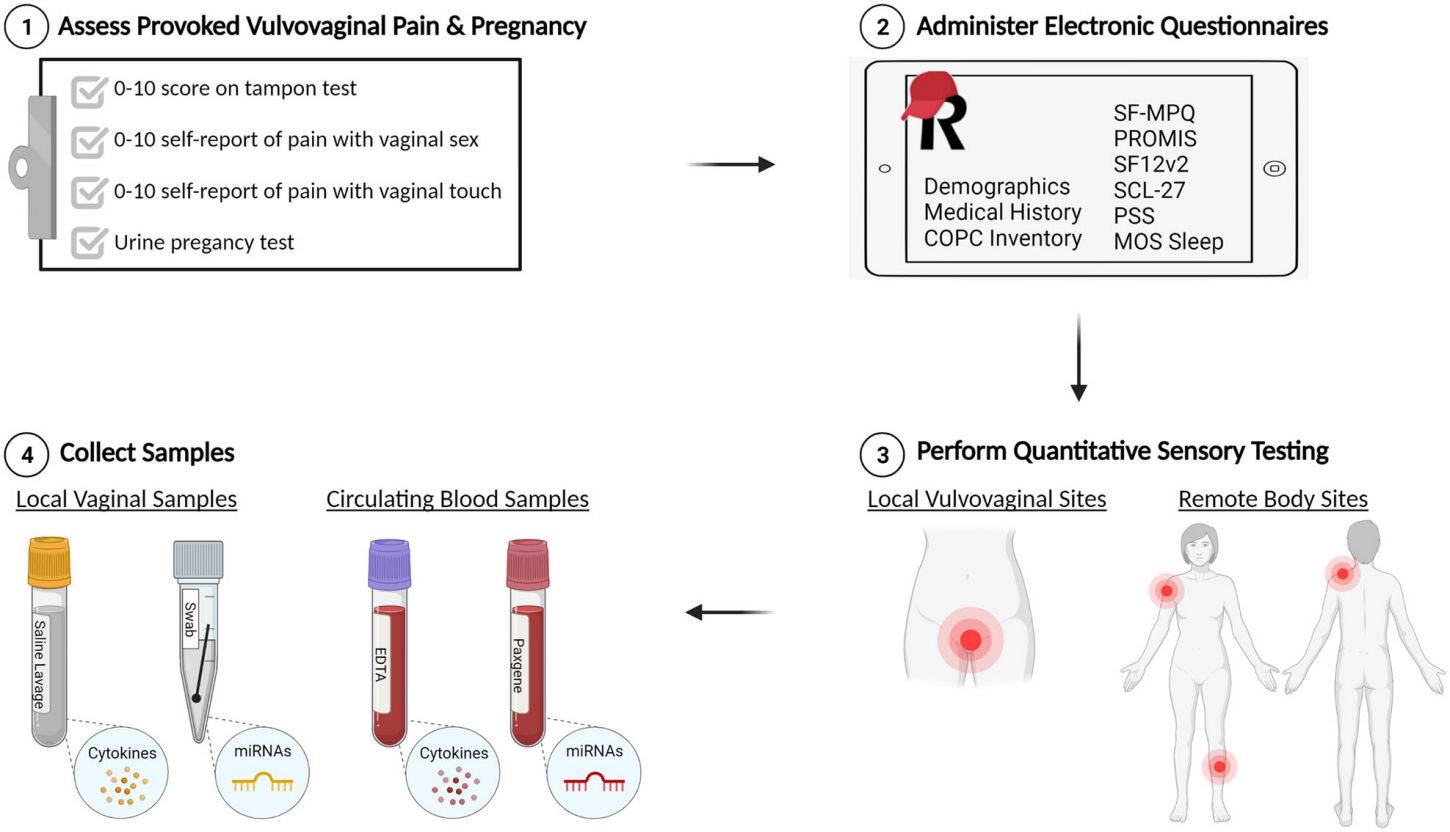
Study visit overview. All four study visits will consist of four elements: (1) assessment of inclusion criteria related to provoked vulvovaginal pain during the tampon test or by self-report of insertional sexual activity and/or with touch to the vaginal opening, (2) completion of validated questionnaires to measure perceived pain, health, mood, and sexual function, (3) quantitative sensory testing to measure pressure pain thresholds at local and remote body sites, and (4) collection of biologic samples to measure local (vaginal) and systemic (blood) cytokines and miRNAs.

Outcomes measures will be assessed during each of four study visits, including a baseline visit 1 pre-treatment, an 8-week visit 2 and 16-week visit 3 during treatment, and a 24-week visit 4 post-treatment. As illustrated in Figure 1, study visits will consist of four elements: (1) standardized tampon test to measure provoked vulvovaginal pain, (2) validated questionnaires to measure perceived pain, health, mood, and sexual function, (3) quantitative sensory testing to measure pressure pain thresholds at local and remote body sites, and (4) biologic sample collection to measure local (vaginal) and systemic (blood) cytokines and miRNAs. Please refer to Table 2 for a complete list of measures and events scheduled for each study visit in chronological order.

**Table 2.** Study procedures and assessments in chronological order.

Assessment	Visit 1 Baseline (0 wk)	Visit 2 Treatment (8 wk)	Visit 3 Treatment (16 wk)	Visit 4 Post-treatment (24 wk)
Informed consent	X			
Tampon Test	X	X	X	X
Self-reported pain with sex involving vaginal penetration	X	X	X	X
Self-reported pain with touch to vaginal opening	X	X	X	X
Blood collection	X	X	X	X
Participant surveys				
Demographics	X			
Social history	X			
Gynaecologic history	X			
VPAQ	X	X	X	X
PROMIS BPSFS-F	X	X	X	X
COPC Inventory	X			
COPC follow-up		X	X	X
SF-12v2	X	X	X	X
SCL-27	X	X	X	X
PILL	X	X	X	X
PSS	X	X	X	X
MOS-sleep	X	X	X	X
Vaginal sample collection	X	X	X	X
Pelvic sensory testing	X	X	X	X
Remote bodily PPTs	X	X	X	X
Medication kit distribution	X	X		
Medication compliance		X	X	X

*Abbreviations*: VPAQ: Vulvar Pain Assessment Questionnaire; PROMIS BPSFS-F: Patient-Reported Outcomes Measurement Information System Brief Profile Sexual Function and Satisfaction Female; COPC Inventory: Chronic Overlapping Pain Condition Inventory; SF-12v2: Short Form; SCL-27: Symptom Checklist 27; PILL: Pennebaker Index of Limbic Languidness; PSS: Perceived Stress Scale; MOS Sleep: Medical Outcomes Study Sleep Scale.

#### Aim 1: Compare the efficacy of peripheral, central, and combined treatments in alleviating pain among women with VBD-p and VBD-c

Primary outcome measures will be the net change in vaginal vestibule pain following treatment measured using the tampon test and the final score on the Short-Form McGill Pain Questionnaire (SF-MPQ). The tampon test has good reliability, construct validity, and responsiveness and is a recommended outcome measure for VBD clinical trials [30]. The tampon test alone may underestimate severity of pain among some women with vulvodynia [31], though it does allow for an assessment of daily life experience in women with vulvodynia. A cardboard applicator regular size Tampax® tampon (Proctor & Gamble) will be inserted by the participant without lubrication into her vagina and then she will be asked to verbally rate her pain on a scale of 0-10, with 0 meaning no pain and 10 meaning the worst possible pain. The SF-MPQ is comprised of two parts: Pain Rating Index (PRI) and the pain intensity visual analogue scale. The PRI measures sensory qualities of pain using 11 sensory and 4 affective qualities related to pain. Each qualitative pain descriptor is then rated on the 4-point pain intensity scale and added for the final score. SF-MPQ subscales have been successfully used to measure treatment responses in vulvodynia [32].

Secondary outcome measures include changes in pressure pain thresholds (PPTs) measured at the vaginal vestibule, levator ani muscles, and remote body sites. The vaginal vestibule PPTs will be determined using a cotton swab applied to 3 externally-accessed sites (2 and 10 o’clock on the upper vestibule and 6 o’clock on the lower vestibule). Levator muscle complex PPTs will be determined by applying the algometer internally to the right, midline, and left puborectalis levator muscles sites (5, 6, and 7 o’clock). Remote Body PPTs will be determined by applying the Wagner FPXTM algometer to 3 ‘neutral’ non-pelvic body sites (deltoid, shin, and trapezius), right and left, and calculating a composite score.

##### Expected outcomes

(1) For women with VBD-p, peripheral lidocaine/oestradiol treatment will be more effective than placebo or nortriptyline in reducing numeric pain ratings on the tampon test, perceived pain on the SF-MPQ, and pelvic PPTs on the algometer test at week 8 and week 16. (2) For women with VBD-c, centrally-targeted nortriptyline treatment will be more effective than placebo or lidocaine/oestradiol in reducing numeric pain ratings on the tampon test, perceived pain on the SF-MPQ, and pelvic/remote bodily PPTs on the algometer test at week 16. (3) For women with VBD-c, combined peripheral and centrally-targeted treatments will be most effective in reducing pain on primary and secondary outcome measures at week 16. (4) The two VBD subtypes will exhibit differential responses to treatment withdrawal at week 16, such that at the week 24 visit women with VBD-c will exhibit increases in pain after treatment withdrawal compared to women with VBD-p.

#### Aim 2: Compare the efficacy of peripheral, central, and combined treatments in improving patient reported outcomes among women with VBD-p and VBD-c

Primary outcome measures include the net change in perceived sexual health measured using the Patient-Reported Outcomes Measurement Information System Brief Profile Sexual Function and Satisfaction Female (PROMIS BPSFS-F) and perceived physical and mental health measured using the Short form-12 Health Survey (SF-12) at 16 weeks. The PROMIS BPSFS-F (is a 15-item form developed by the NIH that measures 11 domains of biopsychosocial function and includes an assessment of sexual function measures related to intercourse [33]. These data are used to calculate four subdomain scores (global satisfaction with sex life, interest in sexual activity, lubrication, and vaginal discomfort) which are the primary endpoints. The PROMIS has been used to reliably assess sexual dysfunction associated with chronic pelvic pain [34,35]. The SF-12v2 assesses 8 domains: physical functioning, physical roles, bodily pain, global health, vitality, social functioning, emotional roles and mental health using an algorithm based on answers to 12 mental and physical health-related questions [36]. These data are used to calculate two summary component scores (a physical score and a mental health score) which are the endpoints. The SF12v2 has demonstrated good reliability and validity [37] and used to assess physical and mental health in women with vestibulodynia [38].

Secondary outcome measures include net changes in perceived mood and health using 1) Symptom Check List 27 (SCL-27) 2) Pennebaker Index of Limbic Languidness (PILL), 3) Perceived stress scale (PSS), and 4) Medical Outcomes Study Sleep Scale (MOS-Sleep) at 16 weeks; as well as patient reported outcomes at 8 and 24 weeks. The SCL-27 measures six dimensions of psychological symptoms (depressive, dysthymic, vegetative, agoraphobic, social phobia, and mistrust) which are the endpoints. It is well studied with high reliability and strong correlations to pain and has been validated in chronic pain patients [39]. The PILL is used to create a summary score of somatic symptoms (e.g. itchy eyes, dizziness). Symptom frequency is recorded on a five-point Likert scale ranging from ‘never’ to ‘more than once a week’ [40]. PILL scores are highly correlated with pain severity in individuals with chronic pelvic pain and COPCs [8,41,42]. The PSS is a 10-item scale that measures the impact of personal stress on thoughts and feelings [43]. Participants will complete the PSS-10 on a Likert scale with categories ranging from 1 (Never) to 5 (Very often) and total scores (reverse-scoring Items 4, 5, 7, and 8) are summed 10 items for a final score. PSS has good consistency and reliability with a Cronbach’s alpha of 0.78. The MOS-Sleep is a 12-item scale that measures amount of sleep and ease/difficulty of initiating and maintaining sleep [44]. The MOS-Sleep has high internal reliability [45] and has been successfully used to determine sleep outcomes for patients with FMS [46].

##### Expected outcomes

(1) For women with VBD-p, peripheral lidocaine/oestradiol treatment will be more effective than placebo or nortriptyline in improving perceived physical, mental, and sexual health at 8 and 16 weeks. (2) For women with VBD-c, centrally-targeted nortriptyline treatment will be more effective than placebo or lidocaine/oestradiol in improving perceived physical, mental, and sexual health at 16 weeks. (3) For women with VBD-c, combined peripheral and centrally-targeted treatments will be most effective in improving health at 16 weeks. 4) The two VBD subtypes will exhibit differential responses to treatment withdrawal, such that women with VBD-c report decreases in perceived health from 16 to 24 weeks compared to women with VBD-p.

#### Aim 3: Determine cytokine and microRNA biomarkers that predict treatment response among women with VBD-p and VBD-c

Primary outcome measures include vaginal and systemic cytokine levels and vaginal and systemic microRNA levels at 16 weeks. Cytokine expression levels will be measured in vaginal lavage samples and plasma isolated from whole blood. We will use a standard Human MesoScale Discovery multiplex kit to measure 36 inflammatory mediators (4 tier-1 and 32 tier-2). Tier-1 mediators include the pro-inflammatory cytokines IL-1β [47–49], IL-8 [48,50], and IL-17 [51] and anti-inflammatory cytokine IL-1ra [8,49]. These cytokines were selected based on previous associations with VBD case status and symptom severity. Tier-2 mediators include cytokines (e.g. interleukin 6; IL-6), chemokines (e.g. monocyte chemoattractant protein 1; MCP1), and growth factors (e.g. vascular endothelial growth factor; VEGF) that are involved in peripheral and central sensitization, inflammation, and chronic pain [52]. miRNA expression levels will be measured in vaginal swab samples and blood. RNA will be isolated and then shipped to University of Texas Health Genome Sequencing Facility for small RNA library preparation and sequencing.

Secondary outcome measures will be the collection and reporting of cytokine and microRNA levels at 8 weeks and 24 weeks. We will also identify cytokine and microRNA biomarkers at baseline that predict treatment response at 8, 16, and 24 weeks. We hypothesize that these biomarkers may serve as intermediate phenotypes for treatment success in VBD patient subgroups.

##### Expected outcomes

(1) Women with VBD-p will have higher levels of inflammatory mediators in peripheral vaginal samples, while women with VBD-c will have higher levels of inflammatory mediators in circulating blood measured at the baseline visit (0 weeks). (2) Abnormalities in baseline vaginal cytokine and miRNA biomarkers will predict a positive response to peripheral lidocaine/oestradiol treatment, while abnormalities in blood biomarkers will predict a positive response to centrally-targeted nortriptyline or combined treatment. (3) Peripheral treatment will resolve abnormalities in vaginal biomarkers evaluated at 8, 16, and 24 weeks, while central treatment will resolve abnormalities in blood biomarkers evaluated at 16 weeks.

### Feasibility study

An initial feasibility study was conducted with five women at each study site for the baseline (0 week) visit to optimize and standardize performance of the clinical exam, measurement of PPTs, and collection and processing of blood and vaginal samples. These volunteers were recruited *via* an email blast to the UNC and UCLA students and employees, and were not subject to study inclusion/exclusion criteria. Baseline data were reviewed to determine that the sites performed the testing accurately, prior to recruitment and screening of eligible study participants.

### Statistical considerations

#### VBD subtype designation

Women with VBD-c will be distinguished from those with VBD-p based on the presence of a COPC. The Institute of Medicine’s 2011 report, Relieving Pain in America, identified 9 COPCs that predominantly affect women and frequently co-occur with VBD [53]. The presence of a COPC will be determined using the COPC survey. This is an easy-to-complete form including modules with check-box questions and a body mannequin to determine the presence of 9 COPCs: (1) FMS, (2) TMD, (3) back pain, (4) IBS, (5) chronic tension-type headache, (6) chronic migraine, and (7) chronic fatigue syndrome, (8) endometriosis, and (9) interstitial cystitis. Women with VBD and no COPCs will be designated as VBD-p, while women with VBD and ≥1 COPC will be designated as VBD-c. These criteria will be used as a primary method to distinguish between VBD-p and VBD-c in this study.

We, however, acknowledge that the criteria to distinguish VBD subtypes needs further exploration. In particular, it is possible that the optimal cut-off for VBD-c requires more than one COPC to be present. Therefore, as a secondary method to characterize VBD subtypes, we will calculate a continuous COPC ‘centralness’ score defined as the number of COPCs present ranging from 0 to 9. This continuous COPC score will be used to identify the optimal cut-off value between VBD-p and VBD-c in terms of treatment response.

To further characterize the two VBD subtypes, we will also measure generalized pain at ‘neutral’ non-pelvic sites using a quantitative sensory testing approach designed by Dr. Gracely and colleagues [54,55]. The neutral sites are remote from the pelvic region and not used for diagnosing other pain conditions, but reflect an individual’s overall pressure pain sensitivity [56–59]. An algometer will be applied to 3 neutral sites (deltoid muscle, trapezius, and shin), with continuously ascending pressure at a rate of 1 kg/s to a maximum of 10 kg. Participants will indicate when the evoked sensation first becomes painful. A composite pressure pain threshold (PPT) score will be compared between VBD-c and VBD-p subgroups using ANCOVA. We would expect women with VBD-p to have higher PPT scores (less pain) and women with VBD-c to have lower scores (more pain) at these sites.

#### Analysis population

All analyses will be performed using the Intent-to-Treat (ITT) population. The ITT population will include all randomized participants. Participants will be grouped according to their randomized allocation, regardless of whether the allocated therapy was administered or switched.

#### Statistical analysis

The specific analysis techniques that will be used to analyze each of the study aims will be described in the Statistical Analysis Plan (SAP). Any unexpected deviations from the protocol-specified statistical analyses will also be documented in the SAP. As a relationship between oral contraceptive (OCs) use and VBD has been previously reported [60], we plan to perform a sensitivity analysis that includes as a covariate OC use (as binary or continuous) and its interaction with treatment group to investigate its impact. If OC use is not statistically significant in the model, it will be removed from further analyses.

#### Estimation of power and sample size

We are planning to enroll 400 women with VBD, with an estimated attrition rate of 20%. Of note, Salaffi and colleagues found that vulvar pain measured on a 0-10 NRS has standard deviation 2.0 and that 2 points on a 0–10 NRS correspond to a clinically meaningful difference in pain severity [61]. Using these results and assuming the within-subject correlation of 0.5, at a significance level of 0.05, the proposed sample size will provide sufficient power of at least 0.80 to detect differences as small as 0.82 on a 0–10 scale for the overall treatment main effects (3 arms) vs. placebo. To test each individual treatment effect vs. placebo, at a significance level of 0.0167 (Bonferroni-corrected for 3 arms), the proposed sample size can detect differences as small as 0.98 on a 0-10 scale. At a significance level of 0.05, the proposed sample size can detect differences as small as 1.35 and 1.20 on a 0–10 scale for the interaction effect of treatment and each VBD-subtype, given possible splits of 40/60, 50/50, or 60/40.

To calculate power for research question IIIa, we used data from our previous study of biomarkers in VBD patients [8]. Conservatively assuming that VBD-c patients represent 60% (192/320) of the sample and VBD-p patients represent 40% (128/320) of the sample, and adjusting the significance threshold for 4 tier 1 cytokine tests, we will be able to detect mean differences of at least 0.4 SD with the power of 0.84 or greater using a t-test. For IL-8, 0.4 SD translates to approximately 2 pg/mL, and for IL-1ra 0.4 SD translates to 240 pg/mL of the minimal detectable difference. Of note, the mean differences between controls and VBD patients for IL-8 and IL-1ra in our previous study were 4 pg/mL and 300 pg/mL, respectively. Thus, we have sufficient power to detect plausible differences in cytokine levels between the VBD subgroups. A P value of less than 5% will be regarded as significant.

### Data management

#### Overview of data management

The Duke Coordinating Centre (DCC) will have primary responsibility for data management, including the development of data collection systems, data monitoring processes, and data storage and back-up. State-of-the-art technology will be used for the management of the network’s data. The data will be collected in a validated, IRB approved Redcap survey platform. The DCC team of skilled data managers and programmers will design and produce a tailored network system that provides operational efficiency and meaningful reporting of metrics.

#### Electronic case report forms (eCRF)

The DCC team will develop modules, designed in Redcap, that will capture patient responses to questions having to do with their VBD symptoms. The Redcap instruments will include an enrollment and demographics form; forms for recording relevant history, pain symptoms, vulvar exam results, laboratory results, baseline biomarker levels, and other baseline presenting characteristics; follow-up forms for use during regular follow-up visits; forms to track the participant’s clinical course over time; and others that track all related information for patients that meet the trial criteria.

## Discussion

Vestibulodynia is a common vulvar pain disorder that affects millions of women each year and remains ineffectively treated [1]. While numerous studies highlight the heterogeneity of VBD, women are commonly grouped together for the purpose of evaluating the efficacy of therapies that might preferentially only benefit a specific subset. Thus, we will conduct this multicenter RCT to determine the efficacy of peripheral, centrally-targeted, and combined treatments in alleviating pain among women with distinct VBD subtypes. Further, we will evaluate the validity of clinical and biological factors in predicting treatment responses. We hope these findings will inform more streamlined approaches to the treatment of VBD.

This will be the first multi-centre RCT to compare the efficacy of separate or combined treatments for VBD. The two-by-two factorial study design offers advantages over standard RCTs, including the ability to compare multiple interventions with good statistical power and to detect interactions among interventions [62]. Inclusion of placebo controls for the topical lidocaine + oestradiol compound cream, nortriptyline pills, and combined treatments will add rigour to interpretation of findings and allow us to evaluate potential placebo effects. Finally, leveraging resources at multiple sites should lend to faster recruitment rates, a more heterogeneous patient population, and more generalizable results.

This trial has utilized state-of-the-art technology for the development of electronic systems for data management. Use of eCRFs that capture participant responses as well as clinical and biological data will avoid transcription error and thus improve accuracy by using field constraints to limit entry errors. In comparison to paper forms, such electronic systems will be more secure, efficient, and permit real-time data verification, monitoring, and scoring [63].

In addition to the rigorous study design, this trial uses an innovative approach to identify biological mediators in both local vaginal and circulating blood samples collected at multiple time points (prior to, during, and following treatment). Biomarkers are objective indicators of normal biological processes, pathogenic processes, or response to treatments. Biomarkers are routinely used for the diagnosis, treatment, and risk management for many prevalent health conditions. Tests using miRNA biomarkers are now commercially available for diagnosis of cancers [64]. Work by our lab and others has identified local and circulating cytokines and miRNAs associated with VBD subtypes, pain, and perceived health [8,65]. Through this clinical trial, we hope to identify unique cytokine and miRNA biomarkers in vaginal versus systemic blood tissues that predict responses to different treatments among women with VBD-p versus VBD-c subtypes. In addition, we will create a biorepository of additional blood, plasma, and vaginal lavage samples for future analyses.

Despite these strengths, we also anticipate some limitations related to enrollment, retention, and adherence to medication protocols. With any large RCT, recruitment can be a challenge. It is estimated that nearly 20% of trials close without meeting accrual goals [66]. In anticipation of this challenge, we estimated an attrition rate of 20% in our power analysis. Another challenge is adherence to medication protocols. Some women may not take the study cream or pill consistently. Also, some women may not be able to tolerate the side-effects of the topical cream or oral pill. Further there may be entry errors on the participant medication diary. We will verify actual medication usage by performing pill counts and weighing the cream.

In the wake of the COVID-19 pandemic, we have seen this number rise with important social distancing practices in place. In fact, both UCLA and UNC sites were required to pause recruitment and enrollment for a 4-month period from March - June 2020. During this time, we were able to successfully implement televisits for participants already enrolled. These visits included a virtual review of surveys, collection of dependent variables using shipped materials (e.g. standardized tampons for the tampon test), and distribution of study medication kits by mail.

Ultimately, we may find that there is no significant treatment effect of either the cream or pill for one or both VBD subtypes. Such negative findings are equally valuable, however, as they would inform pain management strategies that do not waste patient’s time with ineffective treatments. Finally, it is important to note that while the 3-month duration of pain is used for VBD classification purposes, it is not recommended to delay treatment in women not enrolling in this study who present to healthcare providers with less than 3 months of symptoms.

## Conclusions

Both peripheral and central treatments are commonly used for the treatment of pain in women with VBD, but there is a lack of data from well-powered RCTs. This two-by-two factorial RCT will provide clinicians with scientific evidence of therapeutic efficacy and further characterize VBD subtypes in an effort to direct and optimize future treatment for women suffering from this disease.

## Supplementary Material

Supplemental Material

## Data Availability

Data sharing is not applicable to this article as no new data were created or analyzed in this study.
