# Supplementary material for: Rationale and design of a multicenter randomized clinical trial of vestibulodynia: understanding pathophysiology and determining appropriate treatments (vestibulodynia: UPDATe)
Source: Ann Med. 2022 Oct 21;54(1):2885–97. doi: 10.1080/07853890.2022.2132531 (PMC9624211; doi:10.1080/07853890.2022.2132531)
Supplement: Supplemental Material [file IANN_A_2132531_SM8385.pdf]

UCLA Consent Form

Consent to Participate in a Research Study

Title of Study: Vestibulodynia: Understanding Pathophysiology and Determining Appropriate Treatments (Vestibulodynia: UPDATe)
NIH Grant Number 1R01HD096331-01 Investigators:
Reference Date: 1/27/20

Andrea Nackley, PhD
Duke University
(919) 613-5911
andrea.nackley@duke.edu

Andrea Rapkin, MD
University of California at Los Angeles
(310) 794-7274
arapkin@mednet.ucla.edu

Erin Carey, MD
University of North Carolina
(919) 966-7764
erin_carey@med.unc.edu

Elizabeth Geller, MD
University of North Carolina
(919) 966-4717
egeller@med.unc.edu

Haley Cutting, MS, PA
University of California at Los Angeles
HCutting@mednet.ucla.edu

STUDY SUMMARY:
Andrea Nackley, PhD, Erin Carey, MD, and Elizabeth Geller, MD are conducting a study with Andrea Rapkin, MD and her associates in the Department of OBGYN at UCLA to identify effective treatments for vestibulodynia (pain at the vaginal opening). You'll be in the study for four visits over about 6 months, which will take place here in the UCLA Medical Plaza.

Women in the study will be randomly (like drawing numbers from a hat) assigned to one of 4 treatment groups and have visits that include physical exams, blood and vaginal sample collection, sensory testing, and questionnaires about your health, mood, pain and sexual function.
 There are risks from the study medicines and creams that are described in this document. Some risks of the medicine include drowsiness, dizziness, dry mouth, constipation, weight gain, and headache. Risks of the vaginal cream include sensations of cold temperature at the site and numbness at the site where the cream is applied.
 The risks from the physical exams include pain and discomfort.

WHAT ARE SOME GENERAL THINGS TO KNOW ABOUT RESEARCH STUDIES?
You are being asked to take part in this research study because you have Vestibulodynia. Please read this consent form carefully and take your time when making your decision about whether to participate. When your study doctor or a study staff member talks with you about this consent form, ask that person to explain any words or information that you do not clearly understand. We encourage you to talk with your family and friends before you decide to take part in this research study. The nature of the study, risks, inconveniences, discomforts, and other important information about the study are described in this document.
Please tell the study doctor or a study staff member if you are taking part in another research study.

WHO WILL PROVIDE FUNDING?
The National Institutes of Health (NIH) is paying Dr Nackley and her colleagues and the doctor's research staff to do this study.

WHO WILL BE MY STUDY DOCTOR?
If you decide to take part in the study, Drs Nackley, Rapkin, Carey and Geller will be your study doctors. A study doctor may be in contact with your regular doctor while you are in the study and afterwards, if needed.

WHY IS THIS STUDY BEING DONE?
We are doing this study to identify effective treatments for vestibulodynia (pain at the vaginal opening). You'll be in the study for about 6 months.

HOW MANY PEOPLE WILL TAKE PART IN THIS STUDY?
About 400 women at two university hospitals in the United States will take part in this study, including about 200 women from this site.

WHAT IS INVOLVED IN THE STUDY?
If you agree to be in this study, you will be asked to sign this consent form.
If you remain eligible for the study after screening, you will be randomly (like drawing numbers from a hat) put into one of four possible treatment groups:
1) vaginal cream (5% lidocaine/0.5 mg/ml 0.02% estradiol) + oral nortriptyline pill
2) vaginal cream (5% lidocaine/0.5 mg/ml 0.02% estradiol) + oral placebo pill
3) vaginal placebo cream + oral nortriptyline pill
4) vaginal placebo cream + oral placebo

A placebo is an inactive substance given in the same form as the active medication so that you cannot tell if you are receiving the actual medication or not. There will be a placebo form of the vaginal cream and of the oral pill.
For the nortriptyline, it will be important not to take any medication that may interact with this medication. We will provide you with a list of medications to avoid.

You will be assigned to one of these four treatment groups by chance, like flipping a coin. You will not know which treatment group you are in, so that we can avoid being biased about the results. However we can find out which treatment group you are in if there is an emergency situation.

You'll be in the study for about six months, and have a total of four research visits (baseline visit, 2 months, 4 months, and 6 months). At each visit you will:
1) Insert a tampon into your vagina to measure any associated pain on a scale from 0-10

2) Have a physical exam to measure pain at the vulva (the area surrounding the vaginal opening) and the vaginal muscles and other body sites (muscle at the shoulder, shin, and back)

3) Complete questionnaires to measure pain, health, mood, and sexual function

4) Have a blood test to measure markers of pain and inflammation in your blood stream

5) Have a vaginal exam to place 5 mL (1 teaspoon) of sterile saline (salt water) in the vagina which is then collected to measure markers of pain and inflammation in the vagina

At the baseline visit we will collect your medical history (including treatment you have already received for your disease) and a review of medication(s) you are now taking.

HOW LONG WILL I BE IN THIS STUDY?
You will be in this study for a total of six months. You can choose to stop participating at any time without penalty or loss of any benefits to which you are entitled. However, if you decide to stop participating in the study, we encourage you to talk with your study doctor first.

WHAT ARE THE RISKS OF THE STUDY?
Some of the questions we will ask you as part of this study and the physical examination may make you feel uncomfortable. You may refuse to answer any of the questions, and you may take a break at any time during the study.
If you are to receive the oral Nortriptyline pill it may cause some, all, or none of the side-effects listed below.

Common
• Drowsiness
• Dizziness
• Dry mouth
• Constipation
• Weight gain
• Headache

Less Common
• Blurred vision
• Difficult urination
• Hallucination
• Fast or slow heartbeat
• Difficulty sleeping
• High or low blood sugar
• Feeling anxious/nervous
• Breast enlargement
• Excessive sweating

Rare, but Serious
• Abnormal heart rhythm
• Abnormally low blood pressure
• Abnormal liver function tests
• Deficiency of white blood cells
• Thoughts of suicide
• Serotonin-syndrome (adverse drug reaction)
• Rash/sun sensitivity
• Heart attack or stroke
• Secondary angle-closure glaucoma
• Hair loss
• Confusion

Some commonly prescribed medications and supplements can very rarely lead to abnormal heart rhythm or serotonin syndrome when mixed with our study medication. The researcher will ask you about what medications you take. If you take one of the medications linked to these rare side-effects, the researcher will give you a handout for abnormal heart rhythm (also found here: https://www.mayoclinic.org/diseases-conditions/long-qt-syndrome/symptoms-causes/syc-20352518?p=1) or serotonin syndrome (also found here: https://www.mayoclinic.org/diseases-conditions/serotonin-syndrome/symptoms-causes/syc-20354758) so that you can understand this small but important risk. If you experience any of these symptoms during the study period, you may be instructed to see your primary care doctor or go to the nearest emergency department.

If you are to receive the Lidocaine/estradiol vaginal cream you may experience some, all, or none of the side-effects listed below.

Common
• Sensations of cold temperature at the site

• Numbness at the site the medication is applied

Less Common
• Hives
• Itching
• Rash
• Redness of skin
• Breast pain or tenderness
• Mood changes
• Nausea
• Vaginal discharge
• Changes in menstrual periods
• Bloating
• Fatigue

Rare, but Serious
• Severe allergic reaction
• Severe burning, stinging, or irritation where the medicine was applied

In addition, you may experience unexpected risks and inconveniences associated with the use of the study medications, physical examination, or sample collection (blood draw and vaginal saline). In any research studies where your personal information is being collected and used, there is the potential risk of loss of confidentiality. Every effort will be made to keep your information confidential, however, this cannot be guaranteed.

Other Study Procedure Risks:
Risks of a blood draw include pain, bleeding, bruising, infection, or swelling at the site of puncture, as well as the possibility of dizziness or fainting. These risks will be minimized by using sterile technique as well as trained clinical personnel to perform blood draws. During each blood draw, we will draw a maximum of 25 ml (5 teaspoons) of blood.

In addition, there may be uncommon or previously unknown problems that might occur. You should report any problems you have to the study team immediately.

Reproductive Risks:
The effects of the study drugs on a developing pregnancy or breastfeeding infant in the doses and forms being used are not well understood. In addition, the changes your body undergoes during pregnancy and lactation may affect some of the things we are measuring in this study. Therefore, women who are pregnant, planning a pregnancy, or breastfeeding are not allowed to participate in the study.

If you are a woman who could possibly become pregnant (you have not completed menopause, or you have not had a hysterectomy and/or both tubes and/or both ovaries removed) and you have a partner who is able to father children, you will have a urine pregnancy test and it must be negative for you to continue in this study.

You and your partner must agree to either abstain completely from vaginal intercourse as long as you are taking the study drug, or use an effective method of contraception for the same length of time. Effective methods include (a) partner vasectomy, (b) bilateral tubal ligation, (c) intrauterine devices (IUDs), (d) hormonal methods (birth control pills, implants, injections, patches, vaginal rings), or (e) barrier methods (condoms, diaphragms, cervical caps) with a spermicide. If you are not currently using one these methods, your study doctor will discuss options with you, given your vestibulodynia and any other medical conditions, your personal preferences, and the level of effectiveness required by this study. Because no method of birth control is 100% effective, you should notify your study doctor immediately if you think there is any chance you could be pregnant.

ARE THERE BENEFITS TO TAKING PART IN THE STUDY?
If you agree to take part in this study, there may not be direct medical benefit to you. We hope that the information learned from this study will benefit other people with your condition in the future.

WHAT ALTERNATIVES ARE THERE TO PARTICIPATION IN THIS STUDY?
You do not have to participate in this study. You can get treatment or care for your illness even if you are not in a research study. You have alternatives for treatment or care, such as other oral medications or vaginal cream medications, physical therapy, and cognitive behavioral therapy and vestibulectomy (surgical removal of the vaginal opening tissue called the vestibule).
Please talk to your primary and/or study doctor about these options before you decide to take part in this study.

HOW WILL MY PRIVACY BE PROTECTED?
Your data will be de-identified, meaning it will not be stored with any identifying information including your name, medical record number, social security number, etc. Only members of the research team will have access to individually identifiable data. All data will be stored electronically in a password protected database, and will only be accessed by the research team.

For studies having a federal Certificate of Confidentiality:
A Certificate of Confidentiality to protect the identity of research participants from forced disclosure will be applied to this study. Avoiding 'forced disclosure' means that doctors, researchers, and other staff involved with this medical research study cannot be forced to provide any subjects' identities in any federal, state or local, civil, criminal, administrative, legislative or other legal proceeding. This Certificate of Confidentiality was issued by the National Institutes of Health, a government agency.
For studies that are required to register on clinical trials.gov:
A description of this clinical trial will be available on http:www.ClinicalTrials.gov, as required by U.S. Law. This website will not include information that can identify you. At most, the website will include a summary of the results. You can search this website at any time.

FUTURE RESEARCH: DATA OR SAMPLES
What we learn in this study may be important to other research in the future. We may use the data and/or specimens collected here in future research or share them with other researchers. We will remove any information that may personally identify you from anything that is used or shared in the future. The specimens may be used for genetic testing. Genetic testing looks at the unique information contained inside a person's cells, called DNA. Genetic tests can be used to identify if certain people or groups of people are more likely to suffer from certain diseases or conditions. The data/specimens may be stored indefinitely.
Any specimens will be kept until the sample is used up. You can request destruction of any stored specimens by emailing this request to Dr. Rapkin: arapkin@mednet.ucla.edu, however, if enough identifying information has been removed, we may not be able to locate your data or specimens. Anything that has already been shared cannot be recalled. You should not expect to receive any results from any future research that may use your data or specimens.

WHAT ARE THE COSTS TO ME IF I PARTICIPATE IN THIS STUDY?
The study will pay for the cost of supplying and administering the study drug, and all required study items and services as described in this consent form.

WHAT ABOUT COMPENSATION?
You will be reimbursed $10 per visits 1 and 2 and $20 per visits 3 and 4, for your expenses related to your participation to cover your gas and time. You will also receive a voucher to cover your parking costs.

WHAT IF I AM INJURED?

It is important that you promptly tell the researchers if you believe that you have been injured because of taking part in this study. You can tell the researcher in person or call him/her at the number(s) listed above.

If you are injured as a result of being in this study, UCLA will provide necessary medical treatment. The costs of the treatment may be covered by the University of California or the study sponsor, or billed to you or your insurer just like other medical costs, depending on a number of factors. The University and the study sponsor do not normally provide any other form of compensation for injury. For more information about this, you may call the UCLA Office of the Human Research Protection Program at 310-206-2040 or email participants@research.ucla.edu.

WHAT IF I WANT TO STOP BEFORE MY PART IN THE STUDY IS COMPLETE?
You can withdraw from this study at any time, without penalty. The investigators also have the right to stop your participation at any time. This could be because you have had an unexpected reaction, or have failed to follow instructions, or because the entire study has been stopped. If you withdraw, no new information will be collected but we will use data that has already been collected. When withdrawing from a study, you must return all unused study drug to your study doctor.

NEW FINDINGS
If important new findings come up that might change your decision to be in this study, you will be given information about those findings as soon as possible. If you choose to stay in the study, you may be asked to sign a new version of the consent form.

WHOM DO I CALL IF I HAVE QUESTIONS OR PROBLEMS?
For questions about the study or a research-related injury, or if you have complaints, concerns or suggestions about the research, contact Dr. Rapkin at: (310) 794-7274, or arapkin@mednet.ucla.edu or your Physician Assistant Haley Cutting at: hcutting@mednet.ucla.edu. If you have questions about your rights as a research subject, or you have concerns or suggestions and you want to talk to someone other than the researchers, you may contact the UCLA OHRPP by phone: (310) 206 2040; by email:participants@research.uc.aedu or by mail: Box 951406, Los Angeles, CA 90095-1406.

STATEMENT OF CONSENT
The purpose of this study, the procedures to be followed, the study's risks and benefits have been explained to me. I have been allowed to ask questions, and my questions have been answered to my satisfaction. I have been told whom to contact if I have questions, to talk about problems, concerns, or suggestions related to the research, or to obtain information or offer input about the research. I have read this consent form (or it has been read to me) and I agree to be in this study, with the understanding that I may withdraw at any time.

I have read the information provided above. I have asked all the questions I have at this time. I will be given a copy of this consent form and the Research Participant's Bill of Rights to keep. I will sign a separate form authorizing access, use, creation, or disclosure of health information about me.

1)	Do you consent to this study?	◯ I ACCEPT<br>◯ I DO NOT ACCEPT
2)	Signature of subject	____
3)	Printed Name of Research Subject	__________________________________
4)	Date of Signature of Research Subject	__________________________________
5)	Signature of Research Team Member Who Obtained Consent	____
6)	Printed Name of Research Team Member Who Obtained Consent	__________________________________
7)	Date of Signature of Research Team Member Who Obtained Consent	__________________________________
8)	I give permission to be contacted for future research studies	◯ Yes<br>◯ No

UNC Consent Form

Please complete the survey below.

Thank you!

---

eConsent version approved: 12.07.2019

Consent to Participate in a Research Study
Title of Study: Vestibulodynia: Understanding Pathophysiology and Determining Appropriate Treatments (Vestibulodynia: UPDATe)
Reference Date: 01/27/2020
NIH Grant Number 1R01HD096331-01 Investigators:

Andrea Nackley, PhD
Duke University
(919) 613-5911
andrea.nackley@duke.edu

Andrea Rapkin, MD
University of California at Los Angeles
(310) 794-7274
arapkin@mednet.ucla.edu

Erin Carey, MD
University of North Carolina
(919) 966-7764
erin_carey@med.unc.edu

Elizabeth Geller, MD
University of North Carolina
(919) 966-4717
egeller@med.unc.edu

Debbie Farb, RN, BSN, MPH, IBCLC
Research Nurse
(919) 886-3024
Debbie.Farb@unc.edu

STUDY SUMMARY:
Andrea Nackley, PhD, Erin Carey, MD, and Elizabeth Geller, MD are conducting a study with Andrea Rapkin, MD and her associates in the Department of OBGYN at UCLA to identify effective treatments for vestibulodynia (pain at the vaginal opening). You'll be in the study for four visits over about 6 months, which will take place here at UNC Hospitals in Hillsborough.
Women in the study will be randomly (like drawing numbers from a hat) assigned to one of 4 treatment groups and have visits that include physical exams, blood and vaginal sample collection, sensory testing, and questionnaires about your health, mood, pain and sexual function.
• There are risks from the study medicines and creams that are described in this document. Some risks of the medicine include drowsiness, dizziness, dry mouth, constipation, weight gain, and headache. Risks of the vaginal cream include sensations of cold temperature at the site and numbness at the site where the cream is applied.
• The risks from the physical exams include pain and discomfort.

WHAT ARE SOME GENERAL THINGS TO KNOW ABOUT RESEARCH STUDIES?
You are being asked to take part in this research study because you have Vestibulodynia. Please read this consent form carefully and take your time when making your decision about whether to participate. When your study doctor or a study staff member talks with you about this consent form, ask that person to explain any words or information that you do not clearly understand. We encourage you to talk with your family and friends before you decide to take part in this research study. The nature of the study, risks, inconveniences, discomforts, and other important information about the study are described in this document. Please tell the study doctor or a study staff member if you are taking part in another research study.

WHO WILL PROVIDE FUNDING?
The National Institutes of Health (NIH) is paying Drs Nackley, Rapkin, Carey and Geller and the doctor's research staff to do this study.

WHO WILL BE MY STUDY DOCTOR?
If you decide to take part in the study, Drs Erin Carey and Elizabeth Geller will be your study doctors. Drs. Carey and Geller may be in contact with your regular doctor while you are in the study and afterwards, if needed.

WHY IS THIS STUDY BEING DONE?
We are doing this study to identify effective treatments for vestibulodynia (pain at the vaginal opening). You'll be in the study for about 6 months.

HOW MANY PEOPLE WILL TAKE PART IN THIS STUDY?
About 400 women at two university hospitals in the United States will take part in this study, including about 200 women from this site.

WHAT IS INVOLVED IN THE STUDY?
If you agree to be in this study, you will be asked to sign this consent form.
If you remain eligible for the study after screening, you will be randomly (like drawing numbers from a hat) put into one of four possible treatment groups:
1) vaginal cream (5% lidocaine/0.5 mg/ml 0.02% estradiol) + oral nortriptyline pill
2) vaginal cream (5% lidocaine/0.5 mg/ml 0.02% estradiol) + oral placebo pill
3) vaginal placebo cream + oral nortriptyline pill
4) vaginal placebo cream + oral placebo

A placebo is an inactive substance given in the same form as the active medication so that you cannot tell if you are receiving the actual medication or not. There will be a placebo form of the vaginal cream and of the oral pill. For the nortriptyline, it will be important not to take any medication that may interact with this medication. We will provide you with a list of medications to avoid.

You will be assigned to one of these four treatment groups by chance, like flipping a coin. You will not know which treatment group you are in, so that we can avoid being biased about the results. However, we can find out which treatment group you are in if there is an emergency situation.

You'll be in the study for about six months, and have a total of four research visits (baseline visit, 2 months, 4 months, and 6 months). At each visit you will:
1) Insert and remove a tampon into your vagina to measure any associated pain on a scale from 0-10

2) Have a physical exam to measure pain at the vulva (the area surrounding the vaginal opening) and the vaginal muscles and other body sites (muscle at the shoulder, shin, and back)

3) During the vaginal exam, your verbal responses to the examination will be captured on the study iPad's built-in recording application. The data file will be deleted immediately after your responses are entered into our capturing system.

4) Complete questionnaires to measure pain, health, mood, and sexual function

5) Have a blood test to measure markers of pain and inflammation in your blood stream

6) Have a vaginal exam to place 5 mL (1 teaspoon) of sterile saline (salt water) in the vagina which is then collected to measure markers of pain and inflammation in the vagina

At the baseline visit we will collect your medical history (including treatment you have already received for your disease) and a review of medication(s) you are now taking.

HOW LONG WILL I BE IN THIS STUDY?
You will be in the study for a total of six months. You can choose to stop participating at any time without penalty or loss of benefits when they are entitled. However, if you decide to stop participating in the study, we encourage

WHAT ARE THE RISKS OF THE STUDY?
Some of the questions we will ask you as part of this study and the physical examination may make you feel uncomfortable. You may refuse to answer any of the questions, and you may take a break at any time during the study.
If you are to receive the oral Nortriptyline pill it may cause some, all, or none of the side-effects listed below.

Common
• Drowsiness
• Dizziness
• Dry mouth
• Constipation
• Weight gain
• Headache

Less Common
• Blurred vision
• Difficult urination
• Hallucination
• Fast or slow heartbeat
• Difficulty sleeping
• High or low blood sugar
• Feeling anxious/nervous
• Breast enlargement
• Excessive sweating

Rare, but Serious
• Abnormal heart rhythm
• Abnormally low blood pressure
• Abnormal liver function tests
• Deficiency of white blood cells
• Thoughts of suicide
• Serotonin-syndrome (adverse drug reaction)
• Rash/sun sensitivity
• Heart attack or stroke
• Secondary angle-closure glaucoma
• Hair loss
• Confusion

Some commonly prescribed medications and supplements can very rarely lead to abnormal heart rhythm or serotonin syndrome when mixed with our study medication. The researcher will ask you about what medications you take. If you take one of the medications linked to these rare side-effects, the researcher will give you a handout for abnormal heart rhythm (also found here: https://www.mayoclinic.org/diseases-conditions/long-qt-syndrome/symptoms-causes/syc-20352518?p=1) or serotonin syndrome (also found here: https://www.mayoclinic.org/diseases-conditions/serotonin-syndrome/symptoms-causes/syc-20354758) so that you can understand this small but important risk. If you experience any of these symptoms during the study period, you may be instructed to see your primary care doctor or go to the nearest emergency department.

If you are to receive the Lidocaine/estradiol vaginal cream you may experience some, all, or none of the side-effects listed below.

Common
• Sensations of cold temperature at the site

• Numbness at the site the medication is applied

Less Common
• Hives
• Itching
• Rash
• Redness of skin
• Breast pain or tenderness
• Mood changes
• Nausea
• Vaginal discharge
• Changes in menstrual periods
• Bloating
• Fatigue

• Severe allergic reaction
• Severe burning, stinging, or irritation where the medicine was applied

In addition, you may experience unexpected risks and inconveniences associated with the use of the study medications, physical examination, or sample collection (blood draw and vaginal saline). In any research studies where your personal information is being collected and used, there is the potential risk of loss of confidentiality. Every effort will be made to keep your information confidential, however, this cannot be guaranteed.

Other Study Procedure Risks:

Risks of a blood draw include pain, bleeding, bruising, infection, or swelling at the site of puncture, as well as the possibility of dizziness or fainting. These risks will be minimized by using sterile technique as well as trained clinical personnel to perform blood draws. During each blood draw, we will draw a maximum of 25 ml (5 teaspoons) of blood.

In addition, there may be uncommon or previously unknown problems that might occur. You should report any problems you have to the study team immediately.

Reproductive Risks:

The effects of the study drugs on a developing pregnancy or breastfeeding infant in the doses and forms being used are not well understood. In addition, the changes your body undergoes during pregnancy and lactation may affect some of the things we are measuring in this study. Therefore, women who are pregnant, planning a pregnancy, or breastfeeding are not allowed to participate in the study.

If you are a woman who could possibly become pregnant (you have not completed menopause, or you have not had a hysterectomy and/or both tubes and/or both ovaries removed) and you have a partner who is able to father children, you will have a urine pregnancy test and it must be negative for you to continue in this study.

You and your partner must agree to either abstain completely from vaginal intercourse as long as you are taking the study drug, or use an effective method of contraception for the same length of time. Effective methods include (a) partner vasectomy, (b) bilateral tubal ligation, (c) intrauterine devices (IUDs), (d) hormonal methods (birth control pills, implants, injections, patches, vaginal rings), or (e) barrier methods (condoms, diaphragms, cervical caps) with a spermicide. If you are not currently using one these methods, your study doctor will discuss options with you, given your vestibulodynia and any other medical conditions, your personal preferences, and the level of effectiveness required by this study. Because no method of birth control is 100% effective, you should notify your study doctor immediately if you think there is any chance you could be pregnant.

ARE THERE BENEFITS TO TAKING PART IN THE STUDY?
If you agree to take part in this study, there may not be direct medical benefit to you. We hope that the information learned from this study will benefit other people with your condition in the future.

WHAT ALTERNATIVES ARE THERE TO PARTICIPATION IN THIS STUDY?
You do not have to participate in this study. You can get treatment or care for your illness even if you are not in a research study. You have alternatives for treatment or care, such as other oral medications or vaginal cream medications, physical therapy, and cognitive behavioral therapy and vestibulectomy (surgical removal of the vaginal opening tissue called the vestibule).
Please talk to your primary and/or study doctor about these options before you decide to take part in this study.

HOW WILL MY PRIVACY BE PROTECTED?
Your data will be de-identified, meaning it will not be stored with any identifying information including your name, medical record number, social security number, etc. Only members of the research team will have access to individually identifiable data. All data will be stored electronically in a password protected database, and will only be accessed by the research team.

• For studies having a federal Certificate of Confidentiality:
The Department of Health and Human Services (HHS) has issued a Certificate of Confidentiality to further protect your privacy. With this Certificate, the investigators may not disclose research information that may identify you in any Federal, State, or local civil, criminal, administrative, legislative, or other proceedings, unless you have consented for this use. Research information protected by this Certificate cannot be disclosed to anyone else who is not connected with the research unless:
1) there is a law that requires disclosure (such as to report child abuse or communicable diseases but not for legal proceedings);
2) you have consented to the disclosure, including for your medical treatment; or
3) the research information is used for other scientific research, as allowed by federal regulations protecting research subjects.
Disclosure is required, however, for audit or program evaluation requested by the agency that is funding this project or for information that is required by the Food and Drug Administration (FDA).

You should understand that a Confidentiality Certificate does not prevent you or a member of your family from voluntarily releasing information about yourself or your involvement in this research. If you want your research information released to an insurer, medical care provider, or any other person not connected with the research, you must provide consent to allow the researchers to release it. This means that you and your family must also actively protect your own privacy.
Finally, you should understand that the investigator is not prevented from taking steps, including reporting to authorities, to prevent serious harm to yourself or others.

• For studies that are required to register on clinical trials.gov:
A description of this clinical trial will be available on http:www.ClinicalTrials.gov, as required by U.S. Law. This website will not include information that can identify you. At most, the website will include a summary of the results. You can search this website at any time.

FUTURE RESEARCH: DATA OR SAMPLES
What we learn in this study may be important to other research in the future. We may use the data and/or specimens collected here in future research or share them with other researchers. We will remove any information that may personally identify you from anything that is used or shared in the future. The specimens may be used for genetic testing. Genetic testing looks at the unique information contained inside a person's cells, called DNA. Genetic tests can be used to identify if certain people or groups of people are more likely to suffer from certain diseases or conditions. The data/specimens may be stored indefinitely.
Any specimens will be kept until the sample is used up. You can request destruction of any stored specimens by emailing this request to Dr. Carey: erin_carey@med.unc.edu, however, if enough identifying information has been removed, we may not be able to locate your data or specimens. Anything that has already been shared cannot be recalled. You should not expect to receive any results from any future research that may use your data or specimens.

Potential Risks and the Genetic Information Non-Discrimination Act (GINA): There is a potential risk of loss of confidentiality. Every effort will be made to protect your confidential information, but this cannot be guaranteed. The genetic information obtained as a result of your participation in this research [will/will not] be included in your medical record. Information from which you may be personally identified will be maintained in a confidential, secure location at DUHS, accessible only by authorized members of the study team, and will not be disclosed to third parties except as described in this consent form, with your permission, or as may be required by law.

The Genetic Information Nondiscrimination Act (GINA) is a Federal law that will protect you in the following ways:
- Health insurance companies and group plans may not request genetic information from this research;
- Health insurance companies and group plans may not use your genetic information when making decisions regarding your eligibility or premiums;
- Employers with 15 or more employees may not use your genetic information when making a decision to hire, promote, or fire you or when setting the terms of your employment.

GINA does not protect you against genetic discrimination by companies that sell life insurance, disability insurance, or long-term care insurance. GINA also does not protect you against discrimination based on an already-diagnosed genetic condition or disease.

WHAT ARE THE COSTS TO ME IF I PARTICIPATE IN THIS STUDY?
The study will pay for research-related items and/or services that are provided only because you are participating in the study. These research-related items and/or services are explained in other areas of this consent form.
You or your health plan may be responsible to pay for all the types of items listed below:

• Items and services that would have been provided to you even if you were not in the study
• Health care given during the study as part of your regular care
• Items or services needed to give you study drugs or devices
• Monitoring for side effects or other problems
• Deductibles or co-pays for these items and/or services

WHAT ABOUT COMPENSATION?
You will be reimbursed $10 per visits 1 and 2 and $20 per visits 3 and 4 for your expenses related to your participation to cover your gas and time. You will receive your compensation at your final study visit.

WHAT WILL HAPPEN IF YOU ARE INJURED BY THIS RESEARCH?
All research involves a chance that something bad might happen to you. If you are hurt, become sick, or develop a reaction from something that was done as part of this study, the researcher will help you get medical care, but the University of North Carolina at Chapel Hill has not set aside funds to pay you for any such injuries, illnesses or reactions, or for the related medical care.

If you think you have been injured from taking part in this study, please contact Dr. Carey at erin_carey@med.unc.edu or (919) 966-7764, or Dr. Geller at egeller@med.unc.edu or (919) 966-4717. They will let you know what you should do.

By signing this form, you do not give up your right to seek payment or other rights if you are harmed as a result of being in this study.

WHAT IF I WANT TO STOP BEFORE MY PART IN THE STUDY IS COMPLETE?
You can withdraw from this study at any time, without penalty. The investigators also have the right to stop your participation at any time. This could be because you have had an unexpected reaction, or have failed to follow instructions, or because the entire study has been stopped. If you withdraw, no new information will be collected but we will use data that has already been collected. When withdrawing from a study, you must return all unused study drug to your study doctor.

NEW FINDINGS
If important new findings come up that might change your decision to be in this study, you will be given information about those findings as soon as possible. If you choose to stay in the study, you may be asked to sign a new version of the consent form.

WHOM DO I CALL IF I HAVE QUESTIONS OR PROBLEMS?
For questions about the study or a research-related injury, or if you have complaints, concerns or suggestions about the research, contact your Research Nurse, Debbie Farb at: debbie.farb@unc.edu or Drs. Carey and Geller at: erin_carey@med.unc.edu or egeller@med.unc.edu. To page Drs. Carey and Geller after-hours and on weekends and holidays, please contact the UNC hospital operator at (919) 966-4131. For questions about your rights as a research participant, contact the Duke IRB at (919) 668-5111.

STATEMENT OF CONSENT
The purpose of this study, the procedures to be followed, the study's risks and benefits have been explained to me. I have been allowed to ask questions, and my questions have been answered to my satisfaction. I have been told whom to contact if I have questions, to talk about problems, concerns, or suggestions related to the research, or to obtain information or offer input about the research. I have read this consent form (or it has been read to me) and I agree to be in this study, with the understanding that I may withdraw at any time.

I have read the information provided above. I have asked all the questions I have at this time. I will be given a copy of this consent form. I will sign a separate form authorizing access, use, creation, or disclosure of health information about me.

---

1) Do you consent to this study?

○ I ACCEPT
○ I DO NOT ACCEPT

---

2) Signature of subject

____

---

3) Printed Name of Research Subject

____

---

4) Date of Signature of Research Subject

____

---

5) Signature of Research Team Member Who Obtained Consent

____

6) Printed Name of Research Team Member Who Obtained Consent ________________________________

7) Date of Signature of Research Team Member Who Obtained Consent ________________________________

8) I give permission to be contacted for future research studies
   ◯ Yes
   ◯ No

Phone & Email Screening for VBD Participants at UNC and UCLA

PHONE SCRIPT

Thank you for calling the UCLA / UNC Department of Obstetrics and Gynecology regarding our research study for vestibulodynia. The purpose of this call is to screen interested participants for this study. This call will take about 10-15 minutes. First, I need to ask you some questions about yourself to determine your eligibility for participation. Second, I will tell you a bit about what participants can expect in this study. If you are eligible for the study and you are interested in participating, we will schedule your first study visit.

Let's begin with questions about yourself:

1. Are you female?
2. Are you between the ages of 18 and 50? (inclusive, participant may be 18 or 50)
3. What is your height and weight? (Refer to BMI Chart below)
4. Can you easily speak, understand, and read English?
5. In the past 3 months, have you experienced pain with sexual contact involving penetration into the vagina, pain with touch to the vaginal opening, or pain with tampon insertion? Or have you avoided vaginal sex, touch, and/or tampon use due to the associated pain?

Answers to all of these questions must be "yes" to continue*. If the answer is no to any of these questions, say: Thank you for answering our questions. We appreciate your interest, but due to the responses you provided, you do not qualify for this study. I will not be able to schedule you for a study visit. We anticipate conducting other research studies in the future and would be delighted if you could check back with us. I can also keep your name and number on file if you would like to be contacted for future studies?

*skip to here if answers to all previous questions are "yes."

1. What medications are you currently taking? Are there any other medications you have taken in the past three months?
2. Do you have any history of intolerance or contraindications to the use of lidocaine or local anesthetics?
3. Do you have any history of intolerance or contraindications to the use of topical estrogen therapy?
4. Do you have history of intolerance to nortriptyline?
5. Do you have any of these contraindications to use of nortriptyline?:
    a. Recent (within the past year) heart attack

   b. Active psychotic or suicidal thoughts
   c. Narrow angle closure glaucoma
6. Are you currently in pelvic physical therapy (pelvic PT) treatment?
7. Do you currently have any skin disease on your vulva? Do you currently have any vaginal infection?
8. Have you been told that you currently have a condition called atrophic vaginitis, which is thinning of the skin of the vaginal canal?
9. Have you had previous surgery to remove part of the vagina or vulva?
10. Have you had Botox to the pelvic floor muscles in the past 12 months or pelvic nerve blocks in the past three months?
11. Are you pregnant, or planning on becoming pregnant during the study period (the study will last for six months from the day of your first study visit)? Have you given birth in the past six months? Are you currently breastfeeding/expressing breastmilk, or in the first three months after stopping breastfeeding/expressing breastmilk?
12. Have you had cancer, chemotherapy and/or radiation treatment within the past year?
13. Do you have any untreated medical condition (e.g., renal impairment, significant hematological disease, cardiovascular disease, hepatic insufficiency, neurological disorder, autoimmune disease, or respiratory illness)
14. Do you have any diagnosed medical condition that places you in a clear inflammatory state? For example, Rheumatoid Arthritis, Lupus, Ulcerative Colitis, Crohn's disease, Ehlers-Danlos Syndrome or other inflammatory condition?
15. Are you post-menopausal, defined as no menstrual period for 12 consecutive months?
16. Have you had both ovaries removed?
17. Are you currently enrolled in another clinical trial or planning to enroll in another clinical trial during the course of this trial?

Answers to all of these questions must be "no" to continue*. If the answer is yes to any of these questions, say: Thank you for answering our questions. We appreciate your interest but due to the responses you provided, you do not qualify for this study. I will not be able to schedule you for a study visit. We anticipate conducting other research studies in the future and would be delighted if you could check back with us. I can also keep your name and number on file if you would like to be contacted for future studies?

If the answer is “yes” to the first set of questions and “no” to the second set of questions, say: It appears that you may qualify to participate in this study. If you are still interested, I would like to tell you a little bit about the study.

This is a research study about vestibulodynia, a condition where women feel pain at the opening of the vagina. They feel this pain regularly when there is pressure in the vagina – pressure from using tampons and/or pressure from sexual activity. Vestibulodynia is very common – it affects 1 out of every 6 women of reproductive age.

Through this study, we hope to find out which medications work best to relieve pain from vestibulodynia, and also find out more about what is happening in the body when women have vestibulodynia.

If you choose to be in the study, you will have a total of four study visits over a six-month time period. Each study visit is 2-3 hours long. At the first study visit, you will:

- Read more details about the study, and sign an informed consent document if you decide you want to participate
- Have a urine pregnancy test
- Insert and remove a tampon by yourself privately in the bathroom, and tell the researcher afterwards how much (if any) pain you felt
- Have your blood drawn
- Complete various questionnaires/surveys on an iPad
- Have a physical exam that includes:
    - Your vital signs
    - Sensory testing at the upper back, outer arm, and shin with a special device called an algometer that measures pressure forces
    - Sensory testing at the opening of the vagina with both a cotton swab (Q-tip) and with a special device called an algometer that measures pressure forces
    - A vaginal speculum exam where samples are gently collected from the vagina in two ways – 1) with a cotton swab (Q-tip) and 2) by instilling and then removing less than a tablespoon of sterile saline (salt water).
- Receive two study medications and instructions for how to take them at home.
    - You will be randomized (like drawing a number from a hat) into one of four treatment groups. Each group will receive a different combination of active and/or inactive (placebo) medications to take during the study.

The total compensation for the study is $60, which includes all four study visits. You will receive your compensation at your final study visit. If you do not complete all four study visits, you will receive compensation based on how many visits you do attend.

After hearing about the study would you be interested in participating? (Schedule patient now). We ask every participant to bring a list of all their current medications to their visits. The medical history we will take is quite detailed, and will require the specific name of each current medication. You may bring in all your medication bottles if you prefer.

We look forward to seeing you on (date, time). Would you prefer that we send your appointment reminder by text, email or phone?

If you have questions about your rights as a research subject, please call Office for Protection of Research Subjects at (310) 825-5344 from 8:00 AM to 5:00 PM, Monday to Friday.

Body Mass Index Table

	NORMAL						OVERWEIGHT					OBESE										EXTREME OBESITY														
BMI	19	20	21	22	23	24	25	26	27	28	29	30	31	32	33	34	35	36	37	38	39	40	41	42	43	44	45	46	47	48	49	50	51	52	53	54
HEIGHT (Inches)												BODY WEIGHT (Pounds)																								
58	91	96	100	105	110	115	119	124	129	134	138	143	148	153	158	162	167	172	177	181	186	191	196	201	205	210	215	220	224	229	234	239	244	248	253	258
59	94	99	104	109	114	119	124	128	133	138	143	148	153	158	163	168	173	178	183	188	193	198	203	208	212	217	222	227	232	237	242	247	252	257	262	267
60	97	102	107	112	118	123	128	133	138	143	148	153	158	163	168	174	179	184	189	194	199	204	209	215	220	225	230	235	240	245	250	255	261	266	271	276
61	100	106	111	116	122	127	132	137	143	148	153	158	164	169	174	180	185	190	195	201	206	211	217	222	227	232	238	243	248	254	259	264	269	275	280	285
62	104	109	115	120	126	131	136	142	147	153	158	164	169	175	180	186	191	196	202	207	213	218	224	229	235	240	246	251	256	262	267	273	278	284	289	295
63	107	113	118	124	130	135	141	146	152	158	163	169	175	180	186	191	197	203	208	214	220	225	231	237	242	248	254	259	265	270	278	282	287	293	299	304
64	110	116	122	128	134	140	145	151	157	163	169	174	180	186	192	197	204	209	215	221	227	232	238	244	250	256	262	267	273	279	285	291	296	302	308	314
65	114	120	126	132	138	144	150	156	162	168	174	180	186	192	198	204	210	216	222	228	234	240	246	252	258	264	270	276	282	288	294	300	306	312	318	324
66	118	124	130	136	142	148	155	161	167	173	179	186	192	198	204	210	216	223	229	235	241	247	253	260	266	272	278	284	291	297	303	309	315	322	328	334
67	121	127	134	140	146	153	159	166	172	178	185	191	198	204	211	217	223	230	236	242	249	255	261	268	274	280	287	293	299	306	312	319	325	331	338	344
68	125	131	138	144	151	158	164	171	177	184	190	197	203	210	216	223	230	236	243	249	256	262	269	276	282	289	295	302	308	315	322	328	335	341	348	354
69	128	135	142	149	155	162	169	176	182	189	196	203	209	216	223	230	236	243	250	257	263	270	277	284	291	297	304	311	318	324	331	338	345	351	358	365
70	132	139	146	153	160	167	174	181	188	195	202	209	216	222	229	236	243	250	257	264	271	278	285	292	299	306	313	320	327	334	341	348	355	362	369	376
71	136	143	150	157	165	172	179	186	193	200	208	215	222	229	236	243	250	257	265	272	279	286	293	301	308	315	322	329	338	343	351	358	365	372	379	386
72	140	147	154	162	169	177	184	191	199	206	213	221	228	235	242	250	258	265	272	279	287	294	302	309	316	324	331	338	346	353	361	368	375	383	390	397
73	144	151	159	166	174	182	189	197	204	212	219	227	235	242	250	257	265	272	280	288	295	302	310	318	325	333	340	348	355	363	371	378	386	393	401	408
74	148	155	163	171	179	186	194	202	210	218	225	233	241	249	256	264	272	280	287	295	303	311	319	326	334	342	350	358	365	373	381	389	396	404	412	420
75	152	160	168	176	184	192	200	208	216	224	232	240	248	256	264	272	279	287	295	303	311	319	327	335	343	351	359	367	375	383	391	399	407	415	423	431
76	156	164	172	180	189	197	205	213	221	230	238	246	254	263	271	279	287	295	304	312	320	328	336	344	353	361	369	377	385	394	402	410	418	426	435	443

EMAIL TEXT

Thank you for contacting the UCLA Department of Obstetrics and Gynecology regarding our research study for vestibulodynia. The purpose of this email is to screen interested participants for this study. Please take your time in reading this detailed email.

First, please review these two sets of questions about yourself to determine your eligibility for participation. Second, please review the information below about what participants can expect in this study. If you are eligible for the study and you are interested in participating, you may reply by email or phone, and we will schedule your first visit.

Let's begin with two sets of questions about yourself:

Question Set #1

1. Are you female?
2. Are you between the ages of 18 and 50? (it is okay to be exactly 18 years old or exactly 50 years old)
3. Can you easily speak, understand, and read English?
4. Is your Body Mass Index (BMI) under 40? You can calculate your BMI here: https://www.nhlbi.nih.gov/health/educational/lose_wt/BMI/bmicalc.htm
5. In the past 3 months, have you experienced pain with sexual contact involving penetration into the vagina, pain with touch to the vaginal opening, or pain with tampon insertion? Or have you avoided vaginal sex, touch, and/or tampon use due to the associated pain?

If you answered "yes" to every question in Set #1, continue reading question Set #2 below. If you answered "no" to any of these questions in Set #1, you will not qualify for this study at this time.

Question Set #2

1. What medications are you currently taking?
2. Are there any other medications you have taken in the past three months?

Question Set #3

1. Do you have any history of intolerance or contraindications to the use of lidocaine or local anesthetics?
2. Do you have any history of intolerance or contraindications to the use of topical estrogen therapy?
3. Do you have history of intolerance to nortriptyline?

4. Do you have any of these contraindications to use of nortriptyline?:
    a. Recent (within the past year) heart attack
    b. Active psychotic or suicidal thoughts
    c. Narrow angle closure glaucoma
5. Are you currently in pelvic physical therapy (pelvic PT) treatment?
6. Do you currently have any skin disease on your vulva? Do you currently have any vaginal infection?
7. Have you been told that you currently have a condition called atrophic vaginitis, which is thinning of the skin of the vaginal canal?
8. Have you had previous surgery to remove part of the vagina or vulva?
9. Have you had Botox to the pelvic floor muscles in the past 12 months or pelvic nerve blocks in the past three months?
10. Are you pregnant, or planning on becoming pregnant during the study period (the study will last for six months from the day of your first study visit)? Have you given birth in the past six months? Are you currently breastfeeding/expressing breastmilk, or in the first three months after stopping breastfeeding/expressing breastmilk?
11. Have you had cancer, chemotherapy and/or radiation treatment within the past year?
12. Do you have any untreated medical condition (e.g., renal impairment, significant hematological disease, cardiovascular disease, hepatic insufficiency, neurological disorder, autoimmune disease, or respiratory illness)
13. Do you have any diagnosed medical condition that places you in a clear inflammatory state? For example, Rheumatoid Arthritis, Lupus, Ulcerative Colitis, Crohn's disease, Ehlers-Danlos Syndrome or other inflammatory condition?
14. Are you post-menopausal, defined as no menstrual period for 12 consecutive months?
15. Have you had both ovaries removed?
16. Are you currently enrolled in another clinical trial or planning to enroll in another clinical trial during the course of this trial?

Thanks for answering these questions to see if you are a good fit for the study at this time. A researcher will review your answers, and let you know if you qualify.

If you are unsure of the answer to any of these questions, or would like further clarification on any of these questions, please send us a reply email or call us by phone. We are happy to discuss any part of these questions further.

What to Expect in this Study
This is a research study about vestibulodynia, a condition where women feel pain at the opening of the vagina. They feel this pain regularly when there is pressure in the vagina – pressure from using tampons and/or pressure from sexual activity. Vestibulodynia is very common – it affects 1 out of every 6 women of reproductive age.

Through this study, we hope to find out which medications work best to relieve pain from vestibulodynia, and also find out more about what is happening in the body when women have vestibulodynia.

If you choose to be in the study, you will have a total of four study visits over a six-month time period. Each study visit is 2-3 hours long. At the first study visit, you will:

- Read more details about the study, and sign an informed consent document if you decide you want to participate
- Have a urine pregnancy test
- Insert and remove a tampon by yourself privately in the bathroom, and tell the researcher afterwards how much (if any) pain you felt
- Have your blood drawn
- Complete various questionnaires/surveys on an iPad
- Have a physical exam that includes:
    - Your vital signs
    - Sensory testing at the upper back, outer arm, and shin with a special device called an algometer that measures pressure forces
    - Sensory testing at the opening of the vagina with both a cotton swab (Q-tip) and with a special device called an algometer that measures pressure forces
    - A vaginal speculum exam where samples are gently collected from the vagina in two ways – 1) with a cotton swab (Q-tip) and 2) by instilling and then removing less than a tablespoon of sterile saline (salt water).
- Receive two study medications and instructions for how to take them at home.
    - You will be randomized (like drawing a number from a hat) into one of four treatment groups. Each group will receive a different combination of active and/or inactive (placebo) medications to take during the study.

The total compensation for the study is $60, which includes all four study visits. You will receive your compensation at your final study visit. If you do not complete all four study visits, you will receive compensation based on how many visits you do attend.

We ask every participant to bring a list of all their current medications to their visits. The medical history we will take is quite detailed, and will require the specific name of each current medication. You may bring in all your medication bottles if you prefer.

If you would believe you qualify for this study and would like to enroll, please reply to this email. We will set up a date and time for your first visit, which will include confirmatory screening procedures to verify your eligibility.

Thank you for your time and consideration. We are eager to meet you and appreciate your interest!

If you have questions about your rights as a research subject, please call Office for Protection of Research Subjects at (310) 825-5344 from 8:00 AM to 5:00 PM, Monday to Friday.

Body Mass Index Table

	NORMAL						OVERWEIGHT					OBESE										EXTREME OBESITY														
BMI	19	20	21	22	23	24	25	26	27	28	29	30	31	32	33	34	35	36	37	38	39	40	41	42	43	44	45	46	47	48	49	50	51	52	53	54
HEIGHT (Inches)																BODY WEIGHT (Pounds)																				
58	91	96	100	105	110	115	119	124	129	134	138	143	148	153	158	162	167	172	177	181	186	191	196	201	205	210	215	220	224	229	234	239	244	248	253	258
59	94	99	104	109	114	119	124	128	133	138	143	148	153	158	163	168	173	178	183	188	193	198	203	208	212	217	222	227	232	237	242	247	252	257	262	267
60	97	102	107	112	118	123	128	133	138	143	148	153	158	163	168	174	179	184	189	194	199	204	209	215	220	225	230	235	240	245	250	255	261	266	271	276
61	100	106	111	116	122	127	132	137	143	148	153	158	164	169	174	180	185	190	195	201	206	211	217	222	227	232	238	243	248	254	259	264	269	275	280	285
62	104	109	115	120	126	131	136	142	147	153	158	164	169	175	180	186	191	196	202	207	213	218	224	229	235	240	246	251	256	262	267	273	278	284	289	295
63	107	113	118	124	130	135	141	146	152	158	163	169	175	180	186	191	197	203	208	214	220	225	231	237	242	248	254	259	265	270	278	282	287	293	299	304
64	110	116	122	128	134	140	145	151	157	163	169	174	180	186	192	197	204	209	215	221	227	232	238	244	250	256	262	267	273	279	285	291	296	302	308	314
65	114	120	126	132	138	144	150	156	162	168	174	180	186	192	198	204	210	216	222	228	234	240	246	252	258	264	270	276	282	288	294	300	306	312	318	324
66	118	124	130	136	142	148	155	161	167	173	179	186	192	198	204	210	216	223	229	235	241	247	253	260	266	272	278	284	291	297	303	309	315	322	328	334
67	121	127	134	140	146	153	159	166	172	178	185	191	198	204	211	217	223	230	236	242	249	255	261	268	274	280	287	293	299	306	312	319	325	331	338	344
68	125	131	138	144	151	158	164	171	177	184	190	197	203	210	216	223	230	236	243	249	256	262	269	276	282	289	295	302	308	315	322	328	335	341	348	354
69	128	135	142	149	155	162	169	176	182	189	196	203	209	216	223	230	236	243	250	257	263	270	277	284	291	297	304	311	318	324	331	338	345	351	358	365
70	132	139	146	153	160	167	174	181	188	195	202	209	216	222	229	236	243	250	257	264	271	278	285	292	299	306	313	320	327	334	341	348	355	362	369	376
71	136	143	150	157	165	172	179	186	193	200	208	215	222	229	236	243	250	257	265	272	279	286	293	301	308	315	322	329	338	343	351	358	365	372	379	386
72	140	147	154	162	169	177	184	191	199	206	213	221	228	235	242	250	258	265	272	279	287	294	302	309	316	324	331	338	346	353	361	368	375	383	390	397
73	144	151	159	166	174	182	189	197	204	212	219	227	235	242	250	257	265	272	280	288	295	302	310	318	325	333	340	348	355	363	371	378	386	393	401	408
74	148	155	163	171	179	186	194	202	210	218	225	233	241	249	256	264	272	280	287	295	303	311	319	326	334	342	350	358	365	373	381	389	396	404	412	420
75	152	160	168	176	184	192	200	208	216	224	232	240	248	256	264	272	279	287	295	303	311	319	327	335	343	351	359	367	375	383	391	399	407	415	423	431
76	156	164	172	180	189	197	205	213	221	230	238	246	254	263	271	279	287	295	304	312	320	328	336	344	353	361	369	377	385	394	402	410	418	426	435	443
